# Comparison Study on the Effect of Mesenchymal Stem Cells-Conditioned Medium Derived from Adipose and Wharton’s Jelly on Versican Gene Expression in Hypoxia

**DOI:** 10.52547/ibj.26.3.202

**Published:** 2022-05-22

**Authors:** Maryam Khani, Bernard Burke, Marzieh Ebrahimi, Shiva Irani, Fattah Sotoodehnejad

**Affiliations:** 1 Department of Biology, School of Basic Science, Science and Research Branch, Islamic Azad University, Tehran, Iran;; 2 Centre for Sports, Exercise and Life Sciences, Faculty of Health and Life Sciences Coventry University, Coventry, UK;; 3 Department of Regenerative Biomedicine at Cell Science Research Center, Royan Institute for Stem Cell Biology and Technology, Tehran, Iran

**Keywords:** Adipose tissue, Hypoxia, Mesenchymal stem cells, Wharton’s jelly

## Abstract

**Background:**

Mesenchymal stem cells enhance tissue repair through paracrine effects following transplantation. The versican protein is one of the important factors contributing to this repair mechanism. Using MSC conditioned medium for cultivating monocytes may increase versican protein production and could be a good alternative for transplantation of MSCs. This study investigates the effect of culture medium conditioned by human MSCs on the expression of the versican gene in PBMCs under hypoxia-mimetic and normoxic conditions.

**Methods:**

The conditioned media used were derived from either adipose tissue or from WJ. Flow cytometry for surface markers (CD105, CD73, and CD90) was used to confirm MSCs. The PBMCs were isolated and cultured with the culture media of the MSC derived from either the adipose tissue or WJ. Desferrioxamine and cobalt chloride (200 and 300 µM final concentrations, respectively) were added to monocytes media to induce hypoxia-mimetic conditions. Western blotting was applied to detect HIF-1α protein and confirm hypoxia-mimetic conditions in PBMC. Versican gene expression was assessed in PBMC using RT-PCR. Western blotting showed that the expression of HIF-1α in PBMC increased significantly (*p* < 0.01).

**Results:**

RT-PCR results demonstrated that the expression of the *versican* and *VEGF* genes in PBMC increased significantly (*p* < 0.01) in hypoxia-mimetic conditions as compared to normoxia.

**Conclusion:**

Based on the findings in the present study, the secreted factors of MSCs can be replaced by direct use of MSCs to improve damaged tissues.

## INTRODUCTION

Mesenchymal stem cells are multipotent cells found both in adult and fetal tissues^[1]^. Because of unique regenerative potential, MSCs have great utility for tissue regeneration and repair in conditions such as cardiac anomalies or injury, bone disorders, and metabolic diseases^[2]^. ADSCs secrete a variety of growth factors and cytokines, including bFGF, KGF, TGF-β, HGF, VEGF, IL-6, -7, -11, and -12, which may mediate the wound-healing effect of ADSCs^[3]^. 

MSCs derived from WJ have transplantable features, including *in vitro* expandability, differentiation abilities, immune evasion and immune regulation potentials^[4]^. These cells possess many potential advantages as transplantable cells for the treatment of various diseases such as cancer, chronic liver disease, cardiovascular diseases, nerve, cartilage, and tendon injury^[4]^. The therapeutic application of the WJ-derived stem cells can be ascribed to the regenerative and immunomodulatory potential of these cells^[5]^.

MSCs have an inhibitory effect on immune cells, such as T and B lymphocytes, natural killer and DCs, and ultimately reduce and modulate immune responses^[6]^. MSCs also decrease the levels of inflammatory cytokines, including TNF-α and IL-12, and increase the level of anti-inflammatory cytokines, such as IL-10, in monocytes^[7]^. 

Monocytes have the ability to differentiate into DCs and macrophages in inflamed or injured tissue, and through this capability, they participate in both innate and adaptive immune responses to various diseases. Monocytes play a key role in inflammatory processes, defense against pathogens, and homoeostosis^[8]^. Versican is an ECM proteoglycan that interacts with cells by binding to non-integrin and integrin receptors and to other ECM components that associate with the cell surface. Versican is produced by leukocytes and demonstrates a marked increase in inflammation, indicating its possible function in tissue repair^[9]^. Versican, as a component of the ECM, impacts immunity and inflammation through regulating immune cell trafficking and activation. Versican is emerging as a potential target in the control of inflammation in a number of different diseases^[10]^. Also, versican takes a role in protecting cells from free radical-induced apoptosis. Stable expression of versican or its C-terminal domain significantly decreased H_2_O_2_-induced cellular apoptosis^[11]^. 

Hypoxia induces a wide range of responses in MSCs, by HIF-1 activation. The activation of this transcription factor changes the gene expression profile of MSCs via a complex series of signals^[12]^. The role of versican in immune modulation, free-radical elimination, and tissue regeneration has been emphasized by researchers. The therapeutic effects of MSCs-derived conditioned media have also been confirmed. While the function of hypoxia and induction of HIF-1α on the expression of many genes in MSCs has previously been discussed, the present study, for the first time, evaluates the effect of conditioned media on the expression of the versican gene in PBMCs and in hypoxia.

## MATERIALS AND METHODS

**Culture media and reagents**

FBS, αMEM, DMEM, penicillin-streptomycin, Trypsin/EDTA solution, PBS, and Ficoll were purchased from Gibco, Germany. Desferrioxamine and DMSO were obtained from Sigma-Aldrich, USA. Cobalt chloride was purchased from Merck, Germany. 

**Cell culture**

ADMSCs and WJ MSCs were acquired from the Royan Stem Cell Bank, Tehran, Iran. Cells were grown in 75-ml plastic ﬂasks in αMEM medium (supplemented with 10% FBS, and 100 U/mL of penicillin and 100 µg/mL of streptomycin) and incubated in a CO_2_ incubator (37 °C, 5% CO_2_, and humidiﬁed atmosphere). Cells were passaged at 80% conﬂuence after five-min treatment with Trypsin/EDTA solution at 37 °C, and centrifugation at 400 ×g for 10 min. Old medium was collected by centrifugation at 400 ×g at 37 °C for 5 min and stored in -70 °C. Monocytes were obtained from blood sample as described before^[13]^.

**Immunophenotype analysis**

ADMSCs and WJ MSCs at 80% conﬂuence were trypsinized and suspended in αMEM at a concentration of 2 × 10^6^/ml, then 100 µl of cells was incubated at 4 °C for 60 min with mouse anti-human monoclonal antibodies against CD73, CD90, CD105, and CD34 (as a control negative), all were conjugated with ﬂuorescein isothiocyanate. The cells were washed twice with PBS and then resuspended and analyzed by ﬂow cytometry (FACS-Calibur, USA). The data were analyzed using FlowJo software. 

**PBMC treatment and hypoxia induction**

PBMCs were treated with the culture medium of ADMSCs and WJ MSCs for 24 hours and then treated with COCl_2_ (300 µM) or desferrioxamine (200 µM), which mimics hypoxia by inducing HIF-1 in the normoxic environment, for an additional 24 hours. In the normoxic control group, PBMC were cultured in water vapor saturated atmospheric air supplemented with 5% CO_2_ at 37 °C for 24 hours.

**Evaluation of HIF-1α expression using Western blot analysis**

PBMCs co-cultured with the conditioned media were collected, lysed and 25 or 50 μg of total lysate protein was subjected to SDS-PAGE. The resolved proteins were transferred to PVDF membrane (Millipore, Billerica, MA, USA). The membranes were blocked with 5% skimmed milk (Anchor, Kowloon, Hong Kong) in PBS Tween-20 (PBS containing 0.1% Tween-20) for one hour and probed for one hour with a HIF-1α monoclonal antibody (1:1000; Abcam, UK), or anti-β-actin (1:10,000; Millipore) antibody in PBS Tween-20. The membranes were washed twice with PBS Tween-20 and incubated with mouse horseradish-peroxidase-conjugated secondary antibody (1:100,000 in PBS Tween-20 (Thermo Fisher Scientific, USA) for 24 hours. After membrane washing step, 300 µl of substrate containing 150 µl of Supersignal West Femto luminal/enhancer solution (Thermo Fisher Scientific) and 150 µl of Supersignal West Femto stable peroxide buffer was added until the bands appeared. β-actin band was used as a control, and band densities were analyzed using Image-J software.

**RNA isolation and cDNA synthesis**

Total cellular RNA was prepared by a single-step method using the Trizol reagent kit (Life Technologies, USA). The RNA extracted from PBMCs was resuspended in diethyl pyrocarbonate-treated water and quantified using a Nanodrop ND-1000 (Thermo Fisher Scientific). To synthesize the cDNA, 4 µl (1 μg) of the extracted RNA was mixed with 1 μl of a random hexamer primer (50 µM), and the volume of the reaction was adjusted to 13.5 μl using ddH_2_O. The reaction was incubated at 70 °C for 5 min. Then 5 μl of 5× buffer, 1 μl of dNTPs (10 mM each), 0.5 μl RNasin (40 U/μl), and 1 μl of M-MLV enzyme (200 U/µl; the final volume of 20 μl; Promega, USA) were mixed in a microtube. The reaction was then incubated at 60 °C for 30 min and then terminated by incubation at 70 °C for 5 min.

**qRT-PCR**

Quantiﬁcation of *VEGF* and versican mRNA was performed by qRT-PCR. PCR reactions were performed by combining 2 µl of cDNA with 6 µl of H_2_O, 10 µl of Sybr Green Master Mix (Takara, Korea), and 1 µl (10 pM) of sense and antisense primers (Sina Clone, Iran; Table 1) for versican and *VEGF* cDNAs. The PCR cycles were as follows: an initial denaturation at 95 °C for 10 min, followed by 40 cycles of denaturation at 95 °C for 20, annealing at 60 °C for 20 s, extension at 72 °C for 20 s, and a final extension at 72 °C. β-2 microglobulin gene was selected as the housekeeping gene^[14]^. 

**Statistical analysis**

Data were generated as mean ± SEM of at least three independent experiments and were analyzed by t-test (GraphPad Prism 6 software, San Diego, USA) to assess differences among the groups.

## RESULTS

**Immunophenotype analysis**

**Fig. 1:**
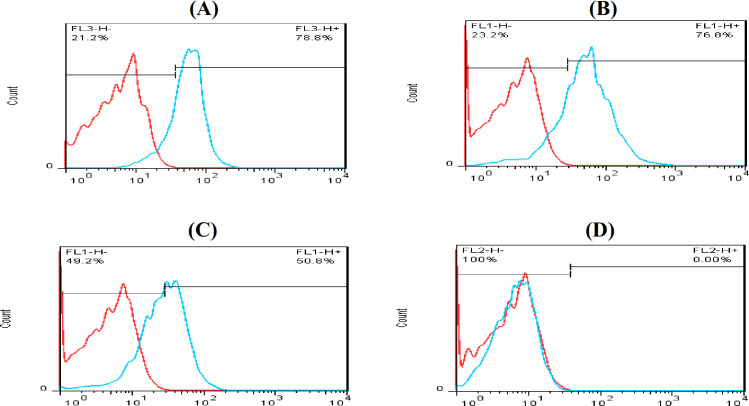
Flow cytometry analysis of ADMSCssurface proteins. Flow cytometry result for (a) CD105 marker on the ADMSCs surface and for CD90 (b), CD73 (c), and (d) CD34, respectively. The red lines reprsent unstain control, and the bule lines represent CD makers expression.

**Fig. 2:**
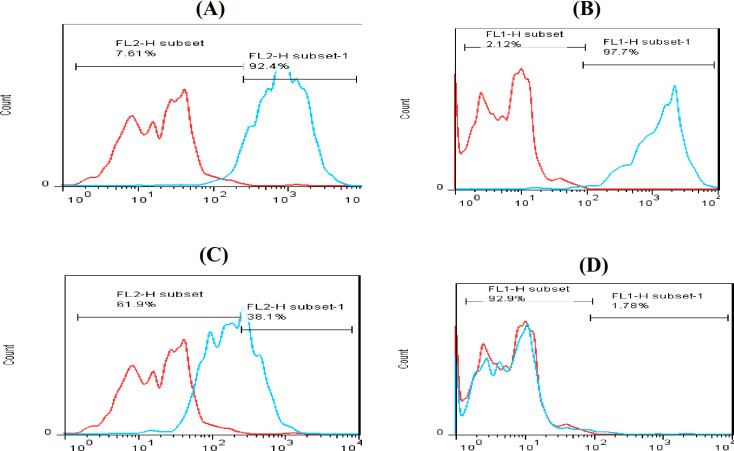
Flow cytometry analysis of WI MSCs surface proteins. Flow cytometry result for (a) CD105 marker on the WJ MSCs surface and for CD90 (b), CD73 (c) and CD34 (D), respectively. The red lines reprsent unstain control, and the bule lines represent CD makers expression.

The presence of ADMSCs was confirmed by the simultaneous expression of the CD90, CD73, and CD105 MSC markers and the lack of expression of the hematopoietic stem cell marker (CD34). The expression of these surface markers was 78.8%, 76.8%, and 50.8% for CD105, CD90, and CD73, respectively, as shown in Figure 1. The results shown in Figure 2 confirmed the presence of WI MSCs. Results also indicated the simultaneous expression of the CD44, CD90, CD73, CD105 (MSCs markers) and the lack of expression of the hematopoietic stem cell marker (CD45). The expression of these surface markers was 89.22%, 92.4%, 97.7%, and 38.1% for CD44, CD105, CD90, and CD73, respectively.

**Evaluation of HIF-1α protein expression**

**Fig. 3:**
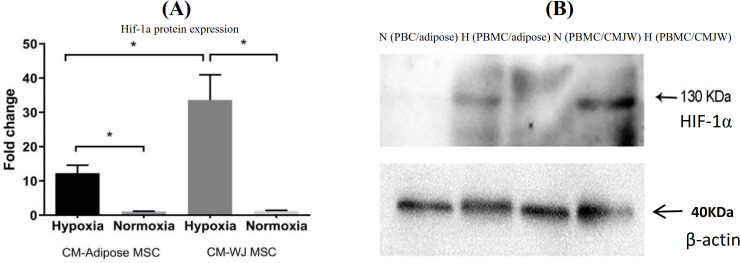
Western blot analysis of HIF-1α protein expression in PBMCs cultured in hypoxia-mimetic or normoxia conditions in the presence of ADMSCs or WJ-derived conditioned media (B). HIF-1α protein expression in hypoxia-mimetic conditions shows a significant increase (*p* < 0.001) compared to normoxia. HIF-1α expression in hypoxia-mimetic conditions after exposure to WJ MSC-conditioned medium was significantly higher (*p* < 0.01) than ADMSCs-conditioned

Western blotting results confirmed hypoxia-mimetic activity in PBMC. The results showed that the expression of HIF-1α protein in the presence of hypoxia mimetics in PBMC cultured with conditioned media increased significantly (*p* < 0.05). Normoxia and HIF-1α expression in WJ MSCs were significantly (*p* < 0.05) higher than ADMSCs in hypoxia-mimetic conditions. As depicted in Figure 3, a HIF-1α protein band (130 kDa) appeared under hypoxia-mimetic conditions. 

**Table 1:** List of primer pairs used

**Gene**	**Primer**	**Sequence **
*β2M*	Forward	GGCTATCCAGCGTACTCCAAAG
	Reverse	CAACTTCAATGTCGGATGGATG
*Versican*	Forward	ACAAGCATCCTGTCTCACG
	Reverse	TGAAACCATCTTTGCAGTGG
*VEGF*	Forward	CAGCGCAGCTACTGCCATCCAATCGAGA
	Reverse	GCTTGTCACATCTGCAAGTACGTTCGTTTA

**Effects of adipose MSC and WJ MSC conditioned media on VEGF and versican gene expression**

**Fig. 4:**
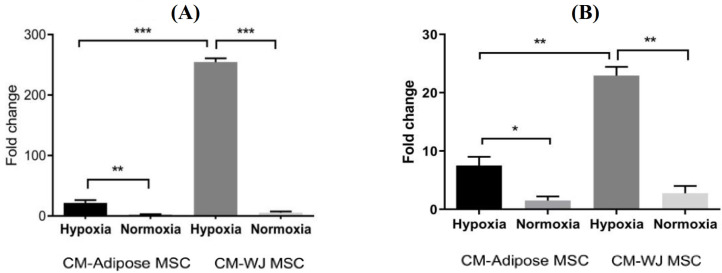
*VEGF* (A) and versican (B) gene expression in hypoxia-mimetic or normoxia conditions in monocytes treated with medium conditioned by either ADMSCs or WJ MSCs. Gene expression was quantified by real-time RT-PCR. In hypoxia-mimetic conditions, the expression of the *VEGF* gene in monocytes significantly increased both in ADMSCs (*p* < 0.01) and WJ MSCs (*p* < 0.001). The expression of the versican gene also increased both in ADMSCs (*p* < 0.05) and WJ MSCs (*p* < 0.01) in hypoxia-mimetic conditions. The data also show that monocyte treatment with WJ MSC-conditioned medium increased the expression of both genes significantly more than the medium of ADMSCs.

The expression of *VEGF* mRNA increased significantly (*p *< 0.01) in PBMCs cultured with the medium conditioned by adipose-MSCs under hypoxia-mimetic conditions as compared to normoxia (Fig. 4). The expression of this gene was even more significantly increased (*p* < 0.001) in PBMC treated with media conditioned by WJ MSCs in hypoxia-mimetic conditions. Similarly, in PBMC treated with adipose MSC-conditioned medium, the versican gene was expressed more significantly (*p* < 0.05) in hypoxia. However, there was a significant enhancement of this effect in PBMC treated with media conditioned by WJ MSCs (*p* < 0.01). The expression of the *VEGF* gene in monocytes significantly increased both in ADMSCs (*p* < 0.01) and WJ MSCs (*p* < 0.001). The expression of the versican gene also increased both in ADMSCs (*p* < 0.05) and WJ MSCs (*p* < 0.01) in hypoxia-mimetic conditions. The data also showed that monocyte treatment with WJ MSC-conditioned medium increased the expression of both genes significantly more than the medium of ADMSCs.

## DISCUSSION

MSCs can be isolated from many human tissues, such as amniotic ﬂuid, bone marrow, muscle, blood, and umbilical cord blood^[15]^. PBMCs respond to signals they receive via their environment; for instance, resident tissue macrophages generate IL-10 in response to activation signals including lipopolysaccharide (LPS)^[16]^. Using the flow cytometry method and studying the surface markers CD105, CD90, and CD73, the identity of the ADMSCs and WJ MSCs were confirmed. Then by evaluating the expression of HIF-1α protein by Western blot analysis, the activity of the hypoxia-mimetic treatment was confirmed in PBMC. RT-PCR results showed that the expression of *VEGF* and versican genes in PBMC cultured with the media of ADMSCs and WJ MSCs exposed to hypoxia condition increased the expression of these two genes. Also, the results displayed that the conditioned medium from WJ MSCs increased the expression of these two genes in monocytes when compared to ADMSCs.

In hypoxia, the expression of two genes (HIF-1α and HIF-2α) increases^[17]^. The binding of HIF-1α and VHL (von-Hippel Lindau) is inhibited, and the HIF-1α protein is not ubiquitylated. As a result, HIF-1α cannot be degraded and accumulates in the cytoplasm, from where it translocates to the nucleus and forms a dimer with HIF-1β, forming the HIF-1 transcription factor. This complex binds to hypoxia response elements (HREs) within the control regions of at least 100 different genes and induces various processes in the cell^[18]^. In a study conducted by Burke *et al.*^[13]^, Western blot analysis was used to compared to the normoxia. Based on the results of this study, the expression of HIF-1α protein in hypoxia significantly increased in monocytes. Shih and Claffey^[19] ^ have shown that *VEGF* gene is affected by the HIF-1 transcription factor. Thus, in hypoxia, the level of *VEGF* gene expression increases. Eming and Krieg^[20]^ have displayed that VEGF and VEGF receptors are important mediators in tissue repair.

Versican is an ECM proteoglycan and one of the genes that respond to hypoxia^[21]^. Research has signified that versican can play a role in the repair of various tissues, including lung, skin, and other tissues^[22,23]^. Reports have highlighted the role of versican in wound healing^[24]^ and in vascular disease, especially atherosclerosis^[25,26]^. Versican binds low-density lipoprotein particles, and accumulation of versican in blood vessel walls is believed to promote extracellular lipoprotein retention and uptake leading to foam cell formation^[27]^. In one study, it was reported that hypoxic induction of versican is regulated by HIF-1 at least in part^[28]^. Also, various studies have pointed to the moderating role of MSCs co-cultured with monocytes and other immune cells *in vitro* and *in vivo*^[29]^. Jiang *et al*.^[30]^ have suggested that human MSCs, when co-cultured with monocytes, prevent the differentiation and function of monocyte-derived DCs, thus modulating the activity of these cells. 

In this study, monocytes cultured with the medium of ADMSCs and WJ MSCs demonstrated an enhanced expression of *VEGF* and versican genes in hypoxia, and the culture medium isolated from WJ MSCs positively affected the expression of these two genes to a greater extent than ADMSCs. Regarding the role of VEGF and versican in tissue repair, and given the overexpression of these two genes under hypoxia, it can be suggested that the factors secreted from this cells may be replaced with direct injection of MSCs to improve tissue repair.

## DECLARATIONS

### Acknowledgments

The financial support of this research by the Science and Research Branch, Islamic Azad University, Tehran, Iran is acknowledged.

### Ethical statement

PBMCs were obtained from blood bags of healthy human donors following obtaining a written informed consent from each subject. The informed consent was prepared and approved by the Review Board at Royan Institute, Tehran, Iran. 

### Data availability

The raw data supporting the conclusions of this article are available from the authors upon reasonable request. 

### Author contributions

MK, drafting of the manuscript and critical revision of the manuscript for important intellectual content; BB, statistical analysis; ME, critical revision of the manuscript for important intellectual content; SI, critical revision of the manuscript for important intellectual content; FS, study concept and design, analysis and interpretation of data, drafting of the manuscript, and statistical analysis.

### Conflict of interest

None declared.

### Funding/support

This research did not receive any specific grant from funding agencies in the public, commercial, or not-for-profit sectors.
